# Cognitive dysfunction following COVID-19 infection

**DOI:** 10.1007/s13365-022-01079-y

**Published:** 2022-05-26

**Authors:** Rafi Hadad, Johad Khoury, Chen Stanger, Tali Fisher, Sonia Schneer, Rachel Ben-Hayun, Katherine Possin, Victor Valcour, Judith Aharon-Peretz, Yochai Adir

**Affiliations:** 1 Neurology, Clalit Health Services, Haifa, Israel; 2 Stroke and Cognition Institute, Rambam Health Care Campus, Haifa, Israel; 3 Global Brain Health Institute (GBHI), University of California San Francisco (UCSF), San Francisco, CA USA; 4 Pulmonology Division, Carmel Medical Center, Haifa, Israel; 5 Pulmonary, Critical Care and Sleep Medicine, Yale University, New Haven, CT USA; 6 Memory and Aging Center, Department of Neurology, University of California San Francisco, San Francisco, CA USA; 7 Ruth and Bruce Rappaport Faculty of Medicine, The Technion, Haifa, Israel

**Keywords:** Cognitive dysfunction following COVID-19 infection, Cognitive decline, Post-COVID, Post-COVID cognitive impairment, Memory symptoms, Executive dysfunction, Cognitive symptoms post-COVID infection

## Abstract

The coronavirus (COVID-19) pandemic is still evolving, causing hundreds of millions of infections around the world. The long-term sequelae of COVID-19 and neurologic syndromes post COVID remain poorly understood. The present study aims to characterize cognitive performance in patients experiencing cognitive symptoms post-COVID infection. Patients evaluated at a post COVID clinic in Northern Israel who endorsed cognitive symptoms were referred for neurologic consultation. The neurologic work-up included detailed medical history, symptom inventory, neurological examination, the Montreal Cognitive Assessment (MoCA), laboratory tests and brain CT or MRI. Between December 2020 and June 2021, 46 patients were referred for neurological consultation (65% female), mean age 49.5 (19–72 years). On the MoCA test, executive functions, particularly phonemic fluency, and attention, were impaired. In contrast, the total MoCA score, and memory and orientation subscores did not differ from expected ranges. Disease severity, premorbid condition, pulmonary function tests and hypoxia did not contribute to cognitive performance. Cognitive decline may affect otherwise healthy patients post-COVID, independent of disease severity. Our examination identified abnormalities in executive function, attention, and phonemic fluency. These findings occurred despite normal laboratory tests and imaging findings.

## Introduction

Coronavirus (COVID-19) disease, often characterized by a severe acute respiratory syndrome following infection with SARS-CoV-2, continues to evolve rapidly. Consequently, the full impact of this infectious disease is not likely to be fully appreciated for years. According to the World Health Organization (WHO), the COVID-19 pandemic has infected more than 298 million people leading to approximately 5.4 million deaths as of late January 2022 (WHO 2022), and it still threatens public health systems worldwide.

Early in the pandemic, attention was focused on acute morbidity and mortality; however, long-term symptoms following infection were reported soon after and these included neuropsychiatric manifestations as well as cognitive issues (Carfi et al. 2020). More than 1 year following the acute stages of infection, many patients report persistent physical and neuropsychiatric symptoms in the aftermath of SARS-CoV-2 infection (WHO 2022; Zubair et al. 2020). Despite emerging evidence that COVID-19 has neurologic consequences, it is yet unclear whether SARS-CoV-2 is neurotropic in humans. Furthermore, the exact mechanism by which SARS-CoV-2 affects the central nervous system (CNS) remains unclear.

During the acute illness, regardless of disease severity, neurologic symptoms have been reported in many COVID-19 patients, including headache, anosmia, ageusia, confusion, encephalopathy, impairment of consciousness, stroke, cerebral venous sinus thrombosis, intracerebral hemorrhage, coma, and seizures (Lau et al. 2006; Madabhavi et al. 2020; Mao et al. 2020; Oxley et al. 2020; Vaira et al. 2020). One-third of patients upon discharge have evidenced cognitive impairment characterized by a dysexecutive syndrome as well as inattention, disorientation, and poorly organized movements in response to commands (Helms et al. 2020; Mao et al. 2020; Poyiadji et al. 2020; Wu et al. 2020).

Previous studies suggest that the presence of anosmia, dysgeusia, diarrhea and the need for oxygen therapy during the acute phase of COVID-19 are risk factors for subsequent cognitive impairment (Almeria et al. 2020). Long-term cognitive complaints are associated with anxiety and depression (Almeria et al. 2020). According to a meta-analysis that used the TriNetX electronic data and evaluated 6-month neurological and psychiatric outcomes among 236,379 patients diagnosed with COVID-19 excluding patients with previous diagnosis of dementia, found that the estimated incidence of a neurological or psychiatric diagnosis 6 months following the infection was 34%, with 13% receiving their first such diagnosis. The most frequent manifestations were vascular complications (e.g., intracranial hemorrhage and ischemic stroke), dementia and psychiatric manifestations (e.g., mood disorders and anxiety). Risk was greatest in, but not limited to, patients who had severe COVID-19 or were hospitalized (Groves and Riley 1988; Hopkins et al. 1999; Mikkelsen et al. 2012).

In the present study, we report the cognitive manifestation of 46 post-COVID patients referred to a community neurology clinic in Northern Israel several months after the diagnosis of COVID-19. We sought to improve the characterization of cognitive impairment of patients recovering from COVID-19 infection.

## Methods

### Participants

This observational study was completed at a post-COVID community clinic in Haifa, Northern Israel. The clinic serves about 1,000,000 citizens residing in north Israel and is affiliated with a 450-bed tertiary care hospital. Patients with a confirmed diagnosis of COVID-19 at least 6 weeks after infection (to allow spontaneous recovery) were questioned about cognitive or neurological symptoms. Those experiencing cognitive symptoms were referred for further neurological evaluation and data were captured for the current study. For the current study, individuals were recruited between December 2020 and June 2021, with a second evaluation 1–7 months after. Forty-six participants were included, 34 were from the Jewish population and 12 were Arabs. All the Jewish participants were fluent in Hebrew except for one who was fluent in English. The Arab participants were fluent in both Arabic and Hebrew.

### Sampling procedures

The study was approved by the local IRB. Participants were first evaluated in the post-COVID community clinic by a pulmonologist and all who reported cognitive symptoms, except those previously diagnosed with dementia or mild cognitive impairment MCI (n = 3) were referred to a neurologist. Those who presented for the neurological appointment were included in the current analysis. All had laboratory confirmed COVID-19 infection.

## Materials

All participants underwent a detailed clinical history completed by the study neurologist (R.H.). Premorbid comorbidities were recorded based on medical records and the assessment during follow-up visit. The evaluation included a physical examination, pulmonary function tests, chest X-ray, and blood tests including a complete blood count, kidney function tests, electrolytes, thyroid function tests, cobalamin B-12 and folic acid levels, and C-reactive protein. The degree of COVID-19 severity was defined as severe or non-severe, based on the Brescia-COVID Respiratory Severity Scale (BCRSS)/Algorithm (score 0 or 1 as non-severe, 2 or 3 for severe cases) (Duca et al. 2020).

The neurological assessment included a detailed history of cognitive symptoms, affective complaints, activities of daily living (ADL), and instrumental activities of daily living (IADL).

The cognitive assessment included a brief screening of global cognitive function, using the Montreal Cognitive Assessment (MoCA) (English, Arabic and Hebrew versions were used, according to the patient native language) (Lifshitz et al. 2012; Rahman and El Gaafary 2009). The MoCA indices reflecting cognitive domains were calculated for each participant as reported in previous studies (Goldstein et al. 2018; Nasreddine et al. 2005).

## Procedure

Cognitive assessments occurred during the first neurological visit. Subsequently, MoCA scores were subdivided into six index scores consistent with prior reports (Table 1) (Kave 2005).

**Table 1:** Description of MoCA indices

**MoCA indices**	Description	Scoring
Memory Index	Number of words recalled in delayed free, category cued and multiple choice, multiplied by 3, 2, and 1, respectively	0–15 points
Executive Index	Trial making, clock, digit span, letter ‘a’ tapping, serial 7 subtraction, phonemic fluency, and abstraction	0–13 points
Visuospatial Index	Cube copy, clock, and confrontational naming	0–7 points
Language Index	Confrontational naming, sentence repetition, and phonemic fluency	0–6 points
Attention index	Digit span, letter ‘a’ tapping, serial 7 subtraction, sentence repetition and words recalled in both immediate recall trials	0–18 points
Orientation Index	All orientation items	0–6 points

For normative group comparisons, we selected the best available normative data available for our patient population, as used in standard clinical practice in the region. The normative sample included 295 cognitively normal participants with a mean age of 73 ± 6 years and a mean educational level was 16.6 ± 2.5 years (Goldstein et al. 2018). Recognizing that this sample is older and has a slightly higher educational attainment level compared to our sample, we note that the comparisons may under-estimate impairment in our study. We used normative data for adult Hebrew speakers for fluency (Kave 2005) and English speakers for MoCA total scores; these normative samples were of similar age and education level to our sample (Rossetti et al. 2011).

## Analytical strategy

### Statistical analysis

Continuous variables were summarized with mean ± SD or median and IQR, categorical variables were presented as numbers and proportions. Kolmogorov–Smirnov test was used to check normality of the different scores. The MoCA index’s scores were compared to those found in the literature for participants with mean age of 72.9 ± 6. Student’s one-tailed t test was applied to test whether the phonemic fluency test’s scores of the COVID patients were significantly lower compared to the Israeli population means. Categorical variables were described as numbers with percentages and compared between groups using Pearson’s chi-squared test. Effect sizes (Cohen’s d) were calculated at the difference between the sample mean and the population mean divided by the population's standard deviation.

Correlation between the different cognitive scores and demographic and clinical characteristics were analyzed using the Spearman correlation for the continuous variables and Mann–Whitney for the categorical variables. The phonemic fluency test scores and the MoCA total scores were converted to Z scores, by matching age and education group (Goldstein et al. 2018; Julayanont et al. 2014; Kave 2005). Statistical analyses were performed using SPSS IBM version 25 (IBM, New York, NY, USA) with a type-I-error (α) set to 0.05 as the statistical threshold for significance.

Post-hoc power analysis of the MoCA indices show that with the study sample size there was at least 90% power to identify a statistically significant difference between the observed and normative performance for any of the indices. Correcting for multiple comparisons using the False Discovery Rate method, the three indices that were statistically significant, Executive, Language, and Attention, remain statistically significant at the p = 0.004 level.

## Results

Between December 2020 and June 2021, 523 participants were evaluated at the post-COVID community clinic, among whom 46 (8%) were also seen at the neurology clinic. Among these, 30 (65%) were females.

The mean age was 50 ± 11.5 years (Table 2) and mean education was 14 ± 3.9 years. Thirty-one participants (67%) had been treated for COVID-19 at home, while 15 (33%) were hospitalized during the acute COVID phase. Thirty-six participants (78%) were diagnosed with non-severe disease. Common comorbidities prior to COVID-19 diagnosis included hyperlipidemia (22%), diabetes mellitus type 2 (20%), asthma (13%), hypertension (13%), Rheumatoid arthritis (4%), as well as COPD, ischemic heart disease, Factor V Leiden deficiency, and familial Mediterranean fever (2% each).

**Table 2:** Demographic data, background and neurologic complains and other symptoms

Demographics	Age	50 ± 11.5
	Female	30 (65%)
	Education years	14 ± 3.9
Comorbidities	Hyperlipidemia	10 (22%)
	DM	9 (20%)
	Hypertension	6 (13%)
	Asthma	6 (13%
	Rheumatoid arthritis	2 (4%)
	Factor V Leiden	1 (2%)
	Familial Mediterranean fever	1 (2%)
	IHD	1 (2%)
	COPD	1 (2%)
Common complaints	Memory symptoms	46 (100%)
	Mood disturbance	38 (84%)
	Dyspnea	36 (80%)
	IADL impairment	31 (67%)
	Cough	30 (67%)
	Headache	27 (60%)
	Arthralgia	13 (29%)
	Hair loss	8 (18%)
	Hyposmia/dysgeusia	7 (15%)
	Hearing loss	4 (9%)
Disease severity	Severe cases	10 (22%)
	Non-severe cases	36 (78%)
	Hospitalized	15 (33%)
	Non-Hospitalized	31 (67%)
Lab Tests	Hemoglobin	13.6 ± 1.4 g/dl
	Cobalamin B12	396 ± 232 pg/mL
	Thyroid stimulating hormone (TSH)	2 ± 0.9 mIU/L
	Forced expiratory flow 1st second (FEV1)	94.2% ± 13.8%
	Forced vital capacity (FVC)	96.2 ± 14.9%
	Diffusion capacity (DLCO)	73.5 ± 12.2%

*IHD* Ischemic Heart Disease, *COPD* Chronic Obstructive Pulmonary Disease, *DM* Diabetes Mellitus, *ADL* Activity of Daily Living

The mean hemoglobin, B12, thyroid stimulating hormone (TSH) were 13.6 ± 1.4 g/dl, 396 ± 232 pg/mL, 2 ± 0.9 mIU/L, respectively. Pulmonary function tests of all patients were reported within the normal range (Table 2). All participants underwent brain imaging with computed tomography (CT), magnetic resonance imaging (MRI) or both, based on clinical needs. These images did not reveal alternative etiologies for the cognitive syndrome (e.g., cerebrovascular disease, inflammatory disorder, or tumor). Five underwent electroencephalography (EEG), which was interpreted as normal in all.

None of the patients were mechanically ventilated; only 33% were hospitalized and required oxygen support. None of the patients were vaccinated prior to the neurological assessment or the follow-up visit, given the time frame of enrollment.

The most frequent self-reported cognitive symptoms were memory symptoms (100%) and mood disturbances (84%). Other frequent symptoms included dyspnea (80%), cough (67%), headache (60%), arthralgia (29%), hair loss (18%) and hearing loss (9%). Thirty-one (67%) reported loss of dependency in IADL due to these cognitive deficits. None reported ADL function impairment.

The mean time between infection and the first ambulatory neurological assessment was 173 ± 80 days. Nineteen participants (41%) had an additional ambulatory follow-up neurological assessment at mean of 302 (± 92) days after infection. Among them, twelve (63%) reported no changes in their cognitive symptoms, two (10%) reported worsening, and five (26%) reported no residual cognitive symptoms. The mean duration of persistent cognitive symptoms was 310 ± 98 days.

**Fig. 1:**
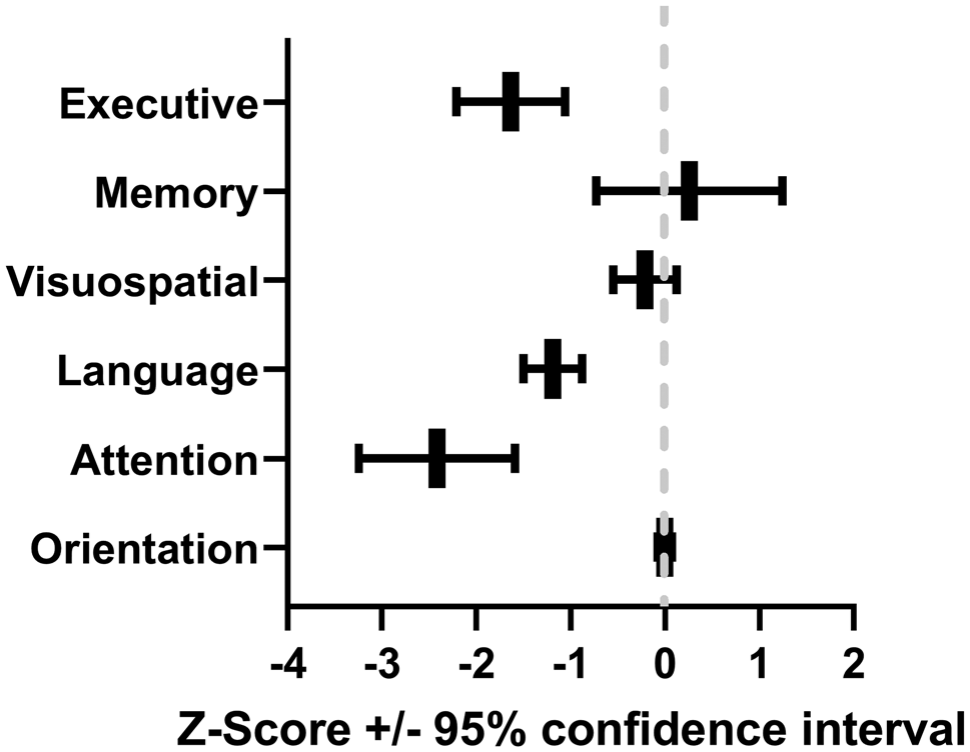
Impact of long COVID syndrome on MoCA index scores

The comparison between our participants’ MoCA index scores versus controls is summarized in Table 3 and Fig. 1. Executive function, language and attention index scores were significantly worse compared to our older normative sample (P < 0.001 for each). In contrast, the memory, visuospatial and orientation index scores did not statistically differ compared to the normative sample. Phonemic fluency, as compared to Israeli controls as previously reported in literature with age and educational attainment adjustment (Lifshitz et al. 2012), we found that our sample had lower z-scores in comparison to the normative data (P < 0.001). The mean z-scores of the phonemic fluency tests were − 1.12 ± 0.82.

**Table 3:** Raw MoCA index scores of participants and normative controls

	Participant performance		Normative performance		95% Confidence interval of the mean difference			
MoCA index (Score range)	Mean	SD	Test value	Mean difference	Lower	Upper	P value	Effect size Cohen’s d
Executive Index (0–13)	10.4	1.9	12.0	−1.6	−2.2	−1.1	< 0.001	1.36
Memory Index (0–15)	10.7	3.3	10.4	0.3	−0.7	1.1	0.600	0.07
Visuospatial Index (0–7)	6.1	1.1	6.3	−0.2	−0.6	0.1	0.200	0.23
Language Index (0–6)	4.3	1.0	5.5	−1.2	−1.50	−0.9	< 0.001	1.60
Attention Index (0–18)	14.5	2.8	17.0	−2.4	−3.2	−1.6	< 0.001	1.80
Orientation Index (0–6)	5.9	0.3	5.9	−0.01	−0.1	0.1	0.888	0.04

The total MoCA score of our patients was not statistically different from controls (P = 0.11). We also found no statistically significant correlation between the MoCA index scores and disease severity, except a trend-level association with the memory index (p = 0.073). Similarly, no associations were found between the MoCA index scores and age, premorbid conditions, time between infection and neurological assessment, self-reporting of mood disturbances, minimal oxygen saturation during the acute phase of COVID-19 and pulmonary function tests at the first visit to the Post COVID-19 clinic (all p-values > 0.10).

## Discussion

In this study uniquely carried out in Northern Israel, we found a characteristic cognitive profile of post-COVID cognitive impairment that involved the specific domains of executive function, language, and attention. In this sample, memory appeared to be preserved when compared to an older normative dataset, suggesting that the self-reported memory symptoms might be due to attentional deficits and executive dysfunction. We noticed a significant deficit in the phonemic fluency testing with greater than 1 SD deviation below that or our normative sample. These findings are similar to recently published studies where patient cognitive symptoms have been described as “Brain Fog” (Graham et al. 2021; Hopkins et al. 1999; Mikkelsen et al. 2012; Zhou et al. 2020). Our study provides confirmation of these findings in a unique northern Israel location, providing evidence for similar patterns globally.”

In this study, the female to male ratio was 2:1 suggesting a possible predominance of cognitive change linked to sex, although factors such as referral bias and bias on those who followed-up for neurological examination cannot be excluded. A predominance of post-COVID-19 cognitive symptoms in women has also been described in previous studies (Graham et al. 2021). According to the literature long-term cognitive impairment and delirium were described in many COVID-19 patients requiring intensive care units (ICU) admission and mechanical ventilation (Graham et al. 2021). We found that age, premorbid conditions, and severity of disease were not statistically significant predictors of long-term cognitive symptoms and dysfunction. In fact, most of our participants were never hospitalized and did not have severe disease as was described in other studies (Graham et al. 2021).

Previous studies note that most of COVID-19 patients fully recover from their cognitive symptoms by 4–6 weeks (Crook et al. 2021; Graham et al. 2021; Greenhalgh et al. 2020; Sudre et al. 2021). In our work, we note that cognitive symptoms persist for more than 10 months among the 14 out of 19 (93%) participants who returned for follow-up. In some cases, these symptoms persisted for more than 1 year.

Due to the limited number of participants who had follow-up neurological assessment, we were unable to have final conclusions about symptoms progression. Further longitudinal studies are needed.

Some participants reported hearing loss (9%). Generally, a hearing assessment confirmed hearing loss without specific etiology noted. Similar complaints have been reported by others (Crook et al. 2021). Further studies are needed to explore the mechanism underlying these complaints. Most of our participants were between 42 and 63 years of age, similar to previous studies (Graham et al. 2021). This age group may be more vulnerable to cognitive impairment after COVID-19 infection; although, this finding might be explained by a referral bias or that some participants didn’t report cognitive symptoms at the post-COVID community clinic.

Attention, memory, verbal fluency, processing speed and executive functioning impairment are well-known complications after severe inflammation of the lungs with multiple mechanisms suspected. One possible mechanism for post-COVID-19 cognitive symptoms is sequel from the cytokine storm associated with the infection (Iwashyna et al. 2010; Lindlau et al. 2015; Mehta et al. 2020; Widmann and Heneka 2014). Most of the participants in our study had mild COVID-19 disease; thus, the severity of acute infection is not likely to be the reason for cognitive symptoms and neuropsychological performance below expectations, at least in most enrollees. Other studies suggest that COVID-19 may promote the pathological accumulation of fibrillar amyloid-β, and can directly induce or aggravate neurodegenerative processes, raising the concern of future dementia (Girard et al. 2018; Heneka et al. 2013; Ising et al. 2019; Sasannejad et al. 2019; Venegas et al. 2017; Wu et al. 2020).

Accordingly, COVID-19 infection may have a continued role in harming brain health until the pandemic ceases.

Our study has several limitations. First, this is a single center study and only 41% of participants attended a follow-up visit. Due to the circumstances of the pandemic, we were not able to enroll a control group and thus are unable to confirm causality. Additionally, the study group was of variable age, background, and disease severity. Due to limitations in the region on normative data, our sample was compared to a group that was dissimilar by ethnic background and age (Goldstein et al. 2018). Despite these caveats, we found statistically lower scores compared to the normative dataset, suggesting that our participants had substantial cognitive deficits in these domains. Our study was performed in a clinical setting. While this strengthens the ecological validity for patients seeking medical care, individuals were referred for diagnostic tests (e.g., imaging, additional laboratory testing) based on clinical indications and not all the individuals received the same tests (e.g., CT, MRI, EEG). Despite this, MRI, CT, EEG were all read to be normal and did not explain the cognitive symptoms.

Unfortunately, the impact of COVID-19 on cognition, and related squeal, diagnosis and possible treatment are still unclear to many clinicians including neurologists. Many of our participants reported difficulty returning to the same work position that they had before COVID-19 infection and 67% reported IADL difficulties. Some had been considered to be malingering in previous assessments despite exhibiting executive dysfunction. Thus, such patients should be diagnosed carefully since normal laboratory and imaging tests may be misleading.

In summary, executive dysfunction, attention disorder and phonemic fluency impairments are seen as long-term sequelae of COVID-19 infection. Our data suggest that reports of memory symptoms may be due to these deficits, since we did not find poor performance in the memory domain. Our data are consistent with prior reports suggesting that females have increased risk for long-term cognitive disorder following COVID-19 infection. In this study, the cognitive deficits were not predicted by age, premorbid conditions, or the severity of COVID-19 disease. Additional systemized studies are needed to track the real impact and long-term sequelae of COVID-19 infection on brain health and cognitive function.
